# Mesenchymal Stem Cells Secretory Responses: Senescence Messaging Secretome and Immunomodulation Perspective

**DOI:** 10.3389/fgene.2017.00220

**Published:** 2017-12-19

**Authors:** Victoria V. Lunyak, Alexandra Amaro-Ortiz, Meenakshi Gaur

**Affiliations:** Aelan Cell Technologies, San Francisco, CA, United States

**Keywords:** senescence, mesenchymal stem cells, SASP, secretome SMS, inflammasome

## Abstract

Mesenchymal stem/stromal cells (MSC) have been tested in a significant number of clinical trials, where they exhibit regenerative and repair properties directly through their differentiation into the cells of the mesenchymal origin or by modulation of the tissue/organ microenvironment. Despite various clinical effects upon transplantation, the functional properties of these cells in natural settings and their role in tissue regeneration *in vivo* is not yet fully understood. The omnipresence of MSC throughout vascularized organs equates to a reservoir of potentially therapeutic regenerative depots throughout the body. However, these reservoirs could be subjected to cellular senescence. In this review, we will discuss current progress and challenges in the understanding of different biological pathways leading to senescence. We set out to highlight the seemingly paradoxical property of cellular senescence: its beneficial role in the development and tissue repair and detrimental impact of this process on tissue homeostasis in aging and disease. Taking into account the lessons from the different cell systems, this review elucidates how autocrine and paracrine properties of senescent MSC might impose an additional layer of complexity on the regulation of the immune system in development and disease. New findings that have emerged in the last few years could shed light on sometimes seemingly controversial results obtained from MSC therapeutic applications.

## Introduction

**Figure 1:**
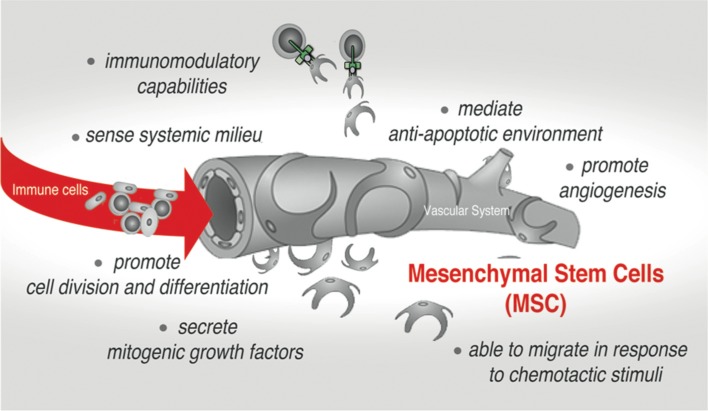
Mesenchymal stem cells (MSC)-mediated effects in native stromal environment and upon therapeutic applications.

Tissue and organ behavior is strongly influenced by the heterogeneous subset of adult mesenchymal stem/stromal cells (MSCs) that reside and can be isolated from almost every type of connective tissues in the adult organism, as well as neonatal tissues including placenta, umbilical cord (UC) and amnion (Uccelli et al., 2008; Hass et al., 2011; Singer and Caplan, 2011). Their developmental origin is still a subject of debate. However it is widely accepted that *embryonic* MSC can be traced to neural crest and neuroepithelium (Takashima et al., 2007; Uccelli et al., 2008), while *adult* MSC are commonly considered to be derived from mural cells (also termed pericytes) residing in the sub-endothelial, perivascular niche (Jiang et al., 2014). The initial enthusiasm of using these cells in regenerative medicine was prompted by a demonstration that MSC can be easily expanded *ex vivo* and have a capacity for differentiation into cells of multiple mesenchymal lineages both *ex vivo* and *in vivo*. Recent studies, however, have redirected the attention of the scientists to yet another remarkable ability of these cells. Much like endothelium and stromal cell, MSC can interact and regulate cells of both the innate and adaptive immune system, triggering several important effector functions in the normal tissue and the pathological settings (Uccelli et al., 2008; Singer and Caplan, 2011; Ben-Ami et al., 2014). Remarkably, after *in vivo* administration and/or in response to endogenous or exogenous damage, MSC can migrate to injured tissue and promote establishment of anti-inflammatory, anti-proliferative, and anti-apoptotic environment, thus fostering both tissue remodeling and survival (Figure 1; Bartholomew et al., 2002; Di Nicola et al., 2002; Chen et al., 2010; Aso et al., 2016; Attar-Schneider et al., 2016). Also, a behavior of cancer cell is strongly affected by the activity of stromal cells, particularly MSC, that are actively recruited into a tumor-associated stromal niche. The current paradigm is that MSC accomplish many of these therapeutically relevant functions *via* a paracrine mechanism. A broad spectrum of secretory factors produced by MSC such as cytokines, chemotactic, ECM remodeling and growth factors has been reported [as reviewed in (Gaur et al., 2017a) and demonstrated in (Ponte et al., 2007; Eggenhofer et al., 2014; Attar-Schneider et al., 2016)].

However, throughout life one can envision that similar to other adult stem cells, changes in the quantity and quality of MSC might influence tissue homeostasis and metabolism, slow down regeneration rate and promote tissue deterioration. Not surprisingly, age-related deficiencies have also been shown to compromise MSC-mediated immunological responses (Signer and Morrison, 2013; Liu et al., 2016). The robust adult stem cell exhaustion is thought to occur due to the process called cellular senescence. Senescence can be inflicted by many intrinsic stimuli, oncogenes, as well as by natural and pathological changes in stem cell microenvironment (Rao and Mattson, 2001; Janzen et al., 2006; Wang et al., 2011; Signer and Morrison, 2013). Indeed, senescence by replicative exhaustion or genotoxic stress during *ex vivo* culturing imposes cell-autonomous and non-cell-autonomous restrictions on MSC. These limitations encompass signaling, metabolic and cytoskeletal changes, which ultimately result in the diminished ability of MSC to cope with DNA damage and other stressors. Reportedly, these changes result in an inability to maintain the structure and function of chromatin, a process indispensable for controlled execution of gene transcription program (Wang et al., 2011; Lopez et al., 2012, 2017).

The emerging evidence suggests that the drawbacks of MSC senescence in tissue and organ homeostasis could be twofold. One of the drawbacks is a loss of tissue repair capacity due to diminishing self-renewal (pool preservation impact) and differentiation (tissue imbalance) caused by the cell cycle arrest. The other is a microenvironment modulation by senescent MSC due to secretion of pro-inflammatory and matrix-degrading molecules, which, if escalated, might have a significant local or systemic impact on overall organism homeostasis. The functional relevance of senescent cells has been reported in three seemingly diverse contexts: (1) in normal embryonic development and regeneration during organ and tissue turnover in adults (beneficial programmed senescence), (2) upon aging and in age-related diseases (harmful chronic senescence), and (3) during therapeutic interventions that deploy potent genotoxic stressors that cause accelerated premature senescence—therapy-induced senescence (TIS; controversially both harmful and beneficial).

Unlike senescence during aging and in age-related disease (discussed elsewhere, Childs et al., 2015; Lasry and Ben-Neriah, 2015), programmed senescence during development and regenerative turnover may be restricted to one or few tissues and organs where MSC are residing. Since MSC are more resistant to programmed apoptosis (Nicolay et al., 2015) and prefer senescent growth arrest to cell death, one can envision that these cells may be the key drivers that potentiate transient, so-called “beneficial senescence” that ensures successful developmental and regenerative outcomes (Munoz-Espin et al., 2013). Contrary to this, many disease-related interventions can induce TIS (Schmitt et al., 2002; Ewald et al., 2010; Nardella et al., 2011; Shao et al., 2013). Senescent MSC can also impose a context-dependent restraint and limitation for numerous therapeutic approaches, one of which is treatment for cancer. While most of the traditional anti-cancer therapies, either generic or targeted, are aimed to induce tumor cell death causing various levels of DNA damage, these therapies might also affect resident MSC. Such resident senescent MSC might exert “bystander” effects inside tumor microenvironment through their capacity to lock immunocompetent cells in a quiescent, non-proliferative state (Uccelli et al., 2008; Rumman et al., 2015) thus helping tumors to evade immune surveillance. In addition, the “bystander” senescent MSC can promote the environment that supports tumor neovascularization and metastasis through the release of angiogenic, migratory, and anti-apoptotic factors (Niu et al., 2015).

In the light of their physiological functions and high therapeutic potential in treatments of numerous diseases such as cancer, tissue injury, and autoimmunity, the understanding of MSC's natural and stressor-induced senescence is of great interest. Limited data are currently available that convincingly demonstrate the impact of cellular senescence caused by exogenous and endogenous stressors on the functional properties of the MSC *in vivo*. However, it is important to factor in the possible influences of senescent MSC on the clinical assessment of novel therapies. TIS is often unpredictable, and that might impose restrictions on immunomodulation properties of endogenous or transplanted MSC and, therefore, interfere with clinical endpoints.

In this review, we will try to provide a synapse of exciting research developments in the field studying different models of senescence *in vivo* and *ex vivo*. We will focus first on uncovering a crosstalk between distinct cellular signaling pathways controlling senescence, and a functional implication of such crosstalk for many processes associated with tissue and organs homeostatic interactions. We will deliberate on numerous studies that brought into focus the ability of senescent cells to communicate with neighboring cells by imposing senescence-specific local microenvironment (niche). We will also discuss the ability of senescence cells to propagate or spread information about their status and build a parallel suggesting how this knowledge can be applied to understanding the functional role of MSC senescence in both non-pathological and pathological settings. Lastly, we will discuss how the senescence cells messaging secretome (SMS) (otherwise known as senescence-associated secretory phenotype, SASP) links together senescence, inflammation, and immunological responses. In this review, we will illustrate examples “when, where, and how” such senescence-orchestrated cascades can be both beneficial and detrimental.

## Senescence: one terminology-many faces

**Figure 2:**
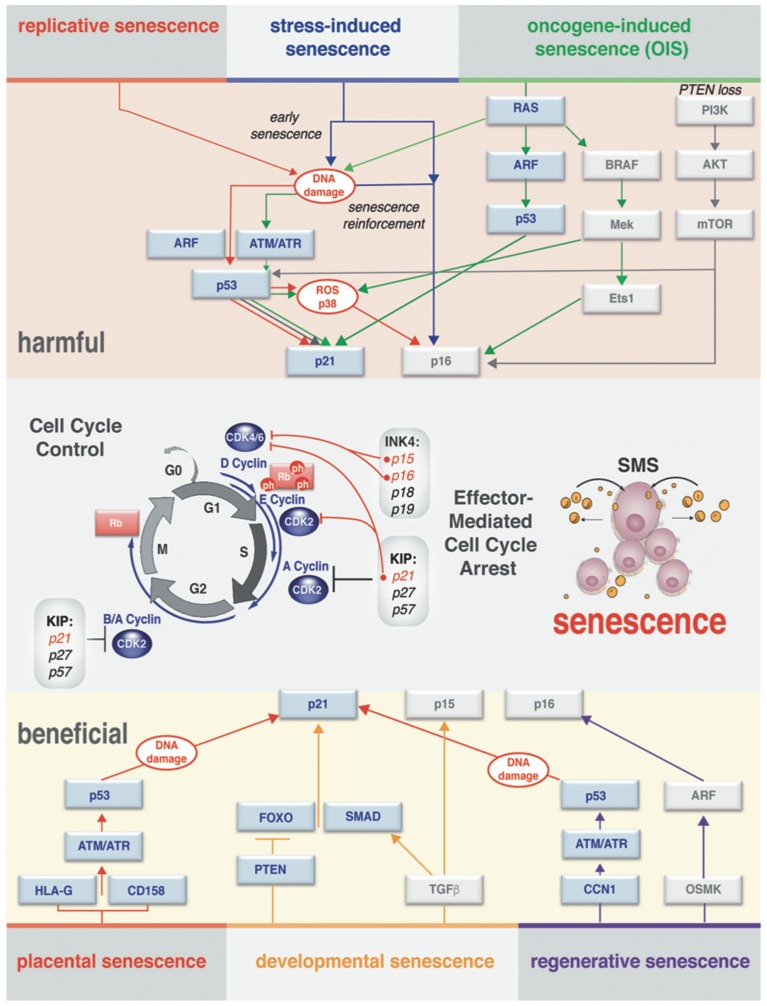
Effector pathways of senescent cell types. Context-dependent induction of the senescence (different senescence modes) observed *in vivo* and *in vitro*. There is a substantial overlap in processing of the stress-response signal and activating effectors of senescence. Both harmful and beneficial senescence in all reported cases results in rising levels of cyclin-dependent kinase inhibitors (CDKI) that drive entry into senescence by activating Rb to block cell cycle progression. Stages of the cell cycle progression and the molecular players through which the cell cycle arrest is executed are outlined in the middle of the diagram. Cell cycle arrest ultimately results in activation of senescence messaging secretome (SMS), which represents a subset of secreted factors broadcasted by senescent cells.

The terminology “*senescent*” describing cells was introduced several decades ago defining a concept that helps to explain the process of maintaining cellular homeostasis with aging. The lessons learned since then indicate that senescence is perhaps not a single unique and unambiguous cell state (Salama et al., 2014; Childs et al., 2015). Overwhelming evidence highlights the fact that distinct triggers can be accountable for physiologically different modes of senescence. Under various endogenous and exogenous stressors, cells engage a distinct, but coordinated network of effector pathways (summarized in Figure 2). Ultimately, these effector pathways converge to exhibit substantial differences in the manifestation of the senescence phenotypes on cell-autonomous and paracrine levels.

Hayflick and colleagues, in the experiments demonstrating that healthy primary cultured cells will exit division cycle after a limited amount of passages (Hayflick and Moorhead, 1961), formally described *replicative senescence*, one of the first characterized senescence modes. Subsequently, it was shown that senescence could be additionally induced prematurely by the activation of oncogenes in primary cells, describing *oncogene-induced senescence* (OIS) (Serrano et al., 1997). This discovery was followed by the notion that in adult cells acute *stress-induced senescence* could be triggered by numerous stress stimuli, including DNA damage, oxidative and metabolic stress, hypoxia, and chemotherapeutic drugs (Schmitt et al., 2002; Narita et al., 2003; Kuilman et al., 2010). Lastly, several new studies have established that induction of senescence can be set and mediated as an integral part of the normal developmental process (*developmental senescence*) by transforming growth factor (TGF)-β/SMAD and PI3K/FOXO pathways (Munoz-Espin et al., 2013; Storer et al., 2013) or pluripotency genes (Chiche et al., 2017).

While the molecular mechanism governing the different type of senescence *in vitro* and *in vivo* is still not fully understood, it is important to make the distinction that senescence could be *transient (acute)* or *chronic*. Such distinction allows grasping the dualistic (either *beneficial* or *harmful)* impact of this process on normal developmental and regenerative events, as well as its role in the pathology of human disease and aging. A summary of the different senescence modes is shown in Figure 2. Next, we will discuss the hallmarks (phenotypic indicators) of senescence that vary according to the nature of the triggers that drive differences in the modes of senescence.

### Replicative senescence

All human somatic and adult stem cells that can be successfully expanded in culture will eventually undergo replicative senescence *in vitro* (Smith and Pereira-Smith, 1996). In proliferating cells, including MSC expanded *ex vivo*, replicative senescence is characterized by a growth arrest, morphological, and cell-size changes, high levels of expression of the tumor suppressors p16^Ink4a^, p21^Cip1^, p53, and/or Rb, and loss of the ability to synthesize and repair DNA (Wang et al., 2011). Within limited population doublings (PD 20-50), cells enlarge, become more granular with increased lysosomal content characterized by senescence-associated β-galactosidase (SA-β-Gal), and slow down their proliferation rate. While resistant to apoptosis, the senescent cells will retain their metabolic activity and can be kept in the culturing media in this state for a prolonged time.

The mechanistic explanation of the underlying replicative cellular senescence process in humans was initially linked to telomere shortening. Telomeres are special DNA structures with repetitive DNA elements which are located at the ends of eukaryotic chromosomes that function to protect the DNA ends from degradation and/or chromosomal ends fusion (Lin et al., 2012). In this point of view, the progressive telomeres erosion associated with replicative divisions is a trigger for initiation of the DNA damage response (DDR). DNA damage sensors ATM/ATR lead to activation and stabilization of p53-dependent checkpoints, which eventually results in the exit from cell cycle mediated by p53-p21^Cip1^ pathway and senescent phenotype (Herbig et al., 2004; Reaper et al., 2004).

However, new data indicate that replicative senescence can also be triggered in human MSC (Wang et al., 2011) and a number of rodent cells prepared from laboratory animals without detectable telomere shortening (Ohtani and Hara, 2013). The appropriate length of the telomeres is maintained by a specialized enzyme, telomerase (Rubtsova et al., 2012). However, in striking contrast to somatic cells, this enzyme is expressed in human germ-line cells and adult stem cells. Also, many somatic rodent cells have telomerase activity (Ohtani and Hara, 2013). This suggests that telomere shortening might not be the only mechanism in play to trigger cellular senescence in rodent and human cells. Recent data have described that replicative senescence of human adult MSC and other somatic cells can be caused by accumulation of unresolved DNA damage in the locations other than telomeres, and activation of persistent DDR pathway coincident with chromatin deterioration due to the loss of epigenetic control and activation of endogenous retrotransposons (Wang et al., 2011; Baker and Sedivy, 2013; De Cecco et al., 2013; Lopez et al., 2017).

Most of the senescent cells have interrupted cell cycle progression (cell cycle arrest) in the G1 phase due to lack of ability to sustain DNA replication. In many cases, this is because the set of cell cycle-dependent kinases (CDK) are inactivated in senescent cells by CDK inhibitors (CDKI) (Sherr and Roberts, 1999) which have been implicated in negative regulation of the cell cycle in p53-dependent and p53-independent manner (Dulic et al., 1994; MacLeod et al., 1995; Hara et al., 1996; Niculescu et al., 1998). Two classes of CDKI have a significant impact on the way cells arrive at cell cycle arrest. First are the KIP/CIP family CDK inhibitors (p21^Cip1^, p27^Kip1^, and p57^Kip2^), which have a dual activity in cell cycle regulation. This family negatively regulates a cell cycle progression by binding to cyclin E, A, B/cdk2, and cyclin D/cdk1 complexes and inhibiting their enzymatic activity (Sherr and Roberts, 1999) as depicted in Figure 2. The second class is INK4 family CDKI (p16^Ink4a^, p15^Ink4b^, p18^Ink4c^, and p19^Ink4d^) that acts upon the cell cycle by blocking the activity of cdk4 and cdk6 and impedes their association with cyclin D (Sherr and Roberts, 1999). Importantly, new findings demonstrate that INK4b-ARF-INK4a locus is playing an important role not only in senescence, but also in stem cell self-renewal (Gil and Peters, 2006). It has been described in mice, that the copy number increase in INK4/ARF and p53 genes by transgenic manipulation of animals extends life span and delays aging by preserving stem cell pool (Carrasco-Garcia et al., 2015).

In normal proliferating cells, there is a very low expression of CDKI (Hara et al., 1996). However, in response to senescence-inducing stimuli, protein expression of the p21^Cip1^ and p16^Ink4a^ genes are drastically increased within the cells (Gil and Peters, 2006). In addition, simultaneous induction of p21^Cip1^ and p16^Ink4a^ cooperatively and efficiently inactivates all CDK, resulting in the Rb-family phosphorylation decline, ultimately triggering senescence cell cycle arrest as illustrated (Figure 2) and reviewed in Ohtani and Hara (2013). Importantly, an elevated intracellular level of another important landmark of senescent cells, reactive oxygen species (ROS), is induced by reciprocal activity of mitogenic signals and components of p16^Ink4a^/Rb-pathway (Takahashi et al., 2006) and Figure 2.

It is commonly accepted that upon replicative senescence, cells enable to replicate DNA due to p21^Cip1^ -imposed block of G1/S-phase transition (Dulic et al., 1994; el-Deiry et al., 1994). However, it is important to acknowledge that p21^Cip1^ (and p27 ^Kip1^) are also involved in G2 cell cycle arrest (Dulic et al., 1998; Niculescu et al., 1998). In this scenario, the cell cycle arrest results in tetraploid senescent cells marked by premature APC/Ccdh1 activation permissive for mitosis skip. Indeed, the stage of cell cycle arrest upon senescence carries important assessment value and particularly matters when DNA content-dependent phenotypic markers are used for the characterization of the mode of senescence such as DNA methylation, the histones content, and histone epigenetic modifications.

### Oncogene-induced senescence

Many cells, including MSC, also undergo oncogene-induced senescence or OIS upon loss of tumor suppressors and/or in response to oncogene activation both *in vitro* and *in vivo* (Braig et al., 2005) and reviewed in Childs et al. (2015). Lowe and colleagues were first to report that in primary cells, the activation of oncogenic mutant rat sarcoma viral oncogene homolog (*Ras*) induces the accumulation of p16^Ink4a^, p53, and ARF and triggers cellular senescence (Serrano et al., 1997). Since then, OIS is increasingly recognized as an effective impediment of cancerous transformations.

*In vivo* activation of oncogenes in response to *Ras, Raf*, and *PTEN* mutations have been reported to lead to senescence in human and mouse tumor models (Braig et al., 2005; Childs et al., 2015). Diverse oncogenic stimuli can drive cells into senescence *via* distinct but interconnected mechanisms: an increased proliferation rate is a landmark of *Ras* activation, where a few rounds of the cellular divisions can cause DNA damage, eventually leading to senescence (D'Adda di fagagna, 2008; Figure 2). Likewise, an activation of *v-raf* murine sarcoma viral oncogene homolog B (BRAF/V600E) has a capacity to induce senescence in both human and mouse melanoma models. This induction, however, occurs through a seemingly different mechanism involving p16^Ink4a^ and activation of pyruvate dehydrogenase (PDH), an essential component of the metabolic signaling axis (Kaplon et al., 2013). An increased mitochondrial metabolism associated with activation of PDH precedes the generation of a high level of ROS within the cells (Figure 2). Data indicate that BRAF-induced senescence could perpetuate upon loss of p16 ^Ink4a^ or p53 activity (Michaloglou et al., 2005; Dhomen et al., 2009; Correia-Melo and Passos, 2015), suggesting that these protein factors are dispensable once senescence is established.

To parallel the oncogene activation, loss of tumor suppressors also potentiates senescence. For instance, abrogation of the tumor suppressor Rb results in senescent phenotype mechanistically dependent on activation of farnesyl-diphosphate synthase and numerous prenyltransferases regulated by sterol regulatory element-binding proteins (SREBPs) (Shamma et al., 2009). This type of senescence antagonizes Rb-deficient carcinogenesis and indirectly leads to the activation of DDR. Another example includes *in vitro* models and in prostate lesions *in vivo*, where the destruction of the tumor suppressor phosphatase and tensin homolog (PTEN) acts as a potent inducer of senescence *via* PI3K –> AKT–> mTOR pathway (Figure 2). This mode of the senescence is sensitive to p53 inactivation (Chen et al., 2005; Alimonti et al., 2010; Wang et al., 2012).

In the context of this discussion it is important to mention that OIS cells often escape proliferative arrest and develop into full-blown malignant cells by three following mechanisms: (1) by acquiring mutation in p53 (Dirac and Bernards, 2003) or p16 ^Ink4a^ (Sage et al., 2003) genes, (2) escape of senescence *via* inactivation of PTEN, leading to the activation of the phosphatidylinositol-4,5-bisphosphate 3-kinase (PI3K) pathway (Vredeveld et al., 2012), or (3) accomplished by chromatin remodeling which bolster an expression of the human telomerase reverse transcriptase gene (hTERT) through activation of c-Myc (Patel et al., 2016). In the later, the telomerase expression resolves existing telomeric DDR foci and extinguishes the DNA replication stress caused by oncogenic signals. Intriguingly, there is also a line of evidence demonstrating that PTEN loss can also become a trigger for senescence that occurs without DNA damage, DDR activation or hyper-replication (Alimonti et al., 2010). These observations suggest that this mechanism might be responsible for triggering senescence in quiescent or terminally differentiated cells.

In MSC, evidence of OIS is sparse and a few reports describe MSC OIS in conjunction with disease manifestation. For instance, leptin and Neutrophil-Activating Peptide 2 (NAP-2) uphold MSC senescence by activation of the PI3K/AKT pathway in patients with systemic lupus erythematosus (Chen H. et al., 2015). Another oncogene, ASPL-TFE3, has also been shown to enable MSC senescence in alveolar soft part sarcoma (Ishiguro and Yoshida, 2016). The field awaits further research on the pathways leading to MSC OIS manifestation in different disease models.

### Stress-induced senescence

**Figure 3:**
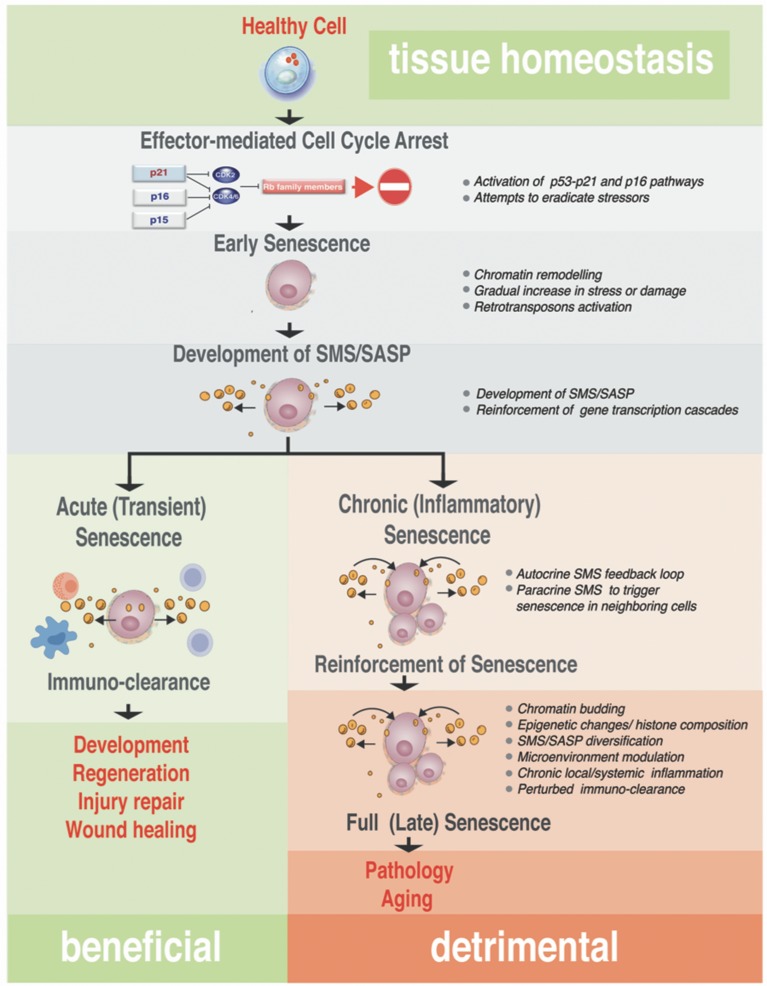
Step-wise processes from acute to chronic senescence. Progressing from early senescence induced by effector-mediated cell cycle arrest either to acute or chronic senescence modes, the diagram shows known biological processes associated with each stage of the senescence (bullets on the right). In all senescence cases, CDKI-mediated licensing of Rb proteins leads to an early senescent stage where the cell cycle arrest still could be reversed by manipulation of a single or combination of factors in the pathway of the action, such as inactivation of p16^Ink4a^ or Rb. These early senescent cells while SA-β-Gal positive may or may not have fully developed SMS. Acute senescence is presumably beneficial, where senescent cells are cleared rapidly by the immune system as part of the program associated with embryonic development, regeneration, wound healing or tissue/organ injury repair. This setting contrasts with chronic senescence, which is a response to the slowly accumulating unresolved macromolecular damage, such as telomere erosion, proteotoxicity, DNA damage, and others. Chronic senescence evolves from acute senescence if immune clearance is impaired with age, leading to prolonged arrest and possibly alterations in the SMS. This type of senescence proceeds to full irreversible senescence through the stage associated with senescence reinforcement driven by cellular changes robust processes, such as epigenetic changes associated with heterochomatinization of cell cycle genes, activation of inflammasome and NF-κB–dependent transcriptional program. Although the full spectrum of drivers of this phenotypic switch *in vivo* is unclear, it likely causes a diversification in SMS that impacts on a local microenvironment and causes local and/or systemic inflammation. This arm of the senescence is detrimental for the tissue and organ homeostasis and probably is an underlying cause of many diseases and pathologies associated with aging.

Prolong cellular stress is another potent inducer of cellular senescence. Potent stressors such as ROS, DNA damage inducing drugs, irradiation and hypoxia have been shown to activate the tumor suppressor p53, resulting in either apoptosis or p21^Cip1^ -driven tansient growth arrest (for details see Childs et al., 2015 and Figure 2). If the cellular repair mechanisms fail to resolve the stress, this transient or acute senescence can build-up to a more prominent senescent phenotype, *via* activation of p16^Ink4a^ that suppresses Rb phosphorylation by inhibition of cdk4 and cdk6 kinases required for Rb phosphorylation (Figure 3). Unphosphorylated Rb proteins bind E2F transcription factors and inhibit them, thus arresting the cell in G1 (Zhang et al., 1999; Bertoli et al., 2013). Existing evidence argues that the cell cycle arrest cannot be revoked by subsequent genetic or pharmacological inactivation of Rb and p53 in this type of cellular senescence. Even if DNA synthesis could be reestablished, the human cells would subsequently fall flat in completing the cell cycle (for details see Ohtani and Hara, 2013; Blagosklonny, 2014).

Interestingly, the studies investigating induction of MSC senescence by oxidative stress, doxorubicin, and bleomycin treatments, as well as irradiation (IR), have outlined the seemingly similar signaling pathways among different stress-inducer stimuli, that confer to the common paracrine circuit that we will discuss later (Ozcan et al., 2016; Gaur et al., 2017b). However, different inducers of senescence promote a preferential use of different metabolic pathways impacting on senescent cells metabolic flexibility (Capasso et al., 2015). Senescent MSC cultures appeared to produce ATP mainly *via* oxidative phosphorylation. Except for irradiated MSC, stress-induced senescent MSC rely primarily on glucose as energy and are unable to freely utilize different energy sources. IR-senescent MSC have shown a lingering ability to use fatty acid and glutamine in cellular metabolic reactions. The study also outlines different levels of ROS production, autophagy and proteasomal activity in different models of MSC stress-induced senescence (Capasso et al., 2015). These data suggest that while stress-specific senescent MSC possess common or shared properties, the differences in the stressor's specific features related to their metabolic activity. This observation is of great importance since metabolism of the stem cells is tightly linked to exhaustion of stem cell compartments and tissue regeneration. In addition, it is an important factor in the setting of either cancer-promoting or restricting microenvironments.

The recently published new study demonstrates that MSC are also sensitive to very low doses of the pesticides generally identified in the food samples (Hochane et al., 2017). The induction of oxidative stress-induced senescence *ex vivo* was reported under this condition. Upon transplantation of senescent MSC in nude mice and subsequent exposure of the animals to the pesticides, the tumorigenic phenotypes were reported in these animals. This data parallels the endogenous properties of senescent MSC described in Lopez et al. (2017) and strongly suggests that exposure to the exogenous stressors can promote the tumorigenic transformation of senescent MSC in the pre-conditioned stromal environment (Hochane et al., 2017).

Interestingly, another form of senescence that could be linked to stress response was also described recently. This mode of senescence is tightly linked to *in vivo* reprogramming of differentiated cells to pluripotent by ectopic expression of transcription factors OCT4, SOX2, KLF4, and cMYC (OSKM) (Mosteiro et al., 2016). Intriguingly, OSKM activity *in vivo* results in two different cellular outcomes. First, OSKM triggers the reprogramming in a small fraction of cells. Second, expression of these four pluripotency master-regulators causes damage and senescence in a much larger population of cells subjected to OSKM forced expression. It comes as no surprise that a hoard of senescent cells observed after injury or in aging was also shown to promote reprogramming (Chiche et al., 2017). Genetic and pharmacological analyses indicate that OSKM-induced senescence requires the INK4a/ARF locus and secretion of interleukin-6 (IL-6), which creates a permissive tissue environment for *in vivo* reprogramming. This type of senescence is indispensable for reprogramming-based tissue repair *in vivo* (Chiche et al., 2017). These new data suggest, that senescence, similar to the programmed apoptosis process (Kourtis and Tavernarakis, 2007; Conradt, 2009; Fuchs and Steller, 2011) might also become imperative during organism development. In development, cellular senescence might occur in a programmed, well-defined manner in response to developmental cues.

### Senescence during embryonic development

Indeed, the evidence of developmentally programmed senescence comes from the studies of mammalian embryogenesis. It was first demonstrated that human placental natural killer (NK) cells undergo senescence *via* p21^Cip1^/DDR signaling pathway and secrete a distinctive specter of cytokines, thus promoting vascular remodeling and angiogenesis in the early stage of the pregnancy (Rajagopalan and Long, 2012; Figure 2). More definitive evidence of the programmed or developmental senescence was observed in studies systematically following senescence-associated markers such as lysosomal β-galactosidase (SA-β-Gal) and CKI during embryogenesis in mice. Senescence, surprisingly, was detectable in many different tissues and locations during particular time windows in the normal developing embryo (Munoz-Espin et al., 2013; Storer et al., 2013). Analysis of microdissected cells from the AER of forelimbs of the mice at E11.5 indicated that the developmental senescence shares a significant similarity in the gene expression signature with OIS. In particular, the commonality was observed in the expression of critical mediators of the senescence program and the paracrine factors (Storer et al., 2013). However, unlike OIS, the developmental senescence is p21^Cip1^ dependent and occurs in the absence of the obvious DNA damage signals and mediators such as ATM/ATR, γH2AX, or p53 activation (Figure 2). No evidence of p19/ARF or p16^Ink4a^ expression was observed in the senescent embryonic tissues, and genetic studies demonstrated that the developmental senescence is p16^Ink4a^ independent (Munoz-Espin et al., 2013). Convincing evidence points to the fact that developmental senescence is mediated by TGF-β/SMAD and PI3K/Forkhead box-O (FOXO) axis (Munoz-Espin et al., 2013; Storer et al., 2013), where TGF-β might also be relevant to the clearance of the senescent cells by macrophages in the absence of widespread detectable apoptosis to continue with developmental program of embryogenesis (Munoz-Espin et al., 2013).

Peculiarly, however, p21^Cip1^-deficient animal models display minimal to no developmental defects (Deng et al., 1995), and genetic studies indicate that the absence of senescence in p21^Cip1^ null mesonephric tubules is compensated by a delayed activation of an apoptotic program that is followed by macrophage-mediated clearance. This research finding indicates that other mechanisms, such as an apoptosis and probably a necrosis, can substitute for senescence-based developmental processes. However, it is important to note that these compensatory processes do not fully replace the absence of developmentally programmed senescence: intriguingly, a failure to undergo senescence does affect adult organ and tissue in a gender-specific manner (Munoz-Espin et al., 2013).

The identification of developmental senescence allowed to put forward a hypothesis that akin to apoptosis during development, developmental senescence is a natural, non-pathological process, which could be later appropriated in the adult as a response to stress (Lasry and Ben-Neriah, 2015).

## Senescence messaging secretome (SMS)

**Figure 4:**
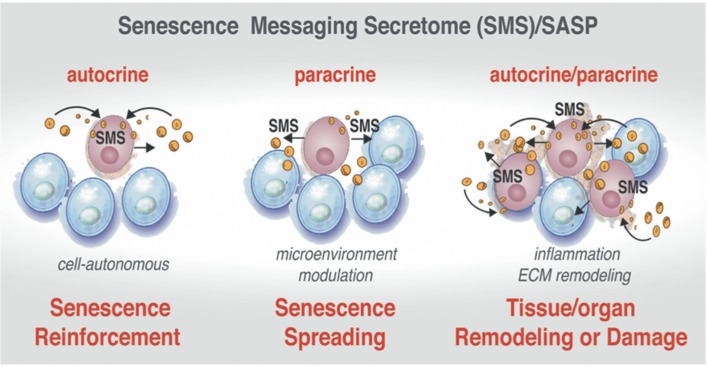
Active communication of the senescent cells with their microenvironment through the SMS. Senescent cells secrete a plethora of factors as a part of senescence messaging secretome (SMS). These factors can reinforce the establishment of the senescence in the cell-of-origin in autocrine manner **(Left)**. The senescent cells can also trigger the microenvironment modulation by acting upon extracellular matrix and on the neighboring cells via secretion of a wide array of the growth factors, cytokines, chemokines, and receptor decoys **(Center)**. SMS also could lead to establishment of senescence in neighboring cells in a paracrine manner **(Right)**. Increasing “senescence footprint” in tissue and organs can lead to further amplification of the SMS and might enhance the transformation of tumor-predisposed cells.

Despite the exit from the cell cycle, the senescent cells in tissues and organs, as well as in development, are not simple passive bystanders but rather very active metabolic players. Numerous studies have provided evidence that the senescence-triggered cellular communication circuitry senescence messaging secretome (SMS) or senescence-associated secretory phenotype (SASP) is necessary for tissue or organ remodeling and regeneration (Coppe et al., 2008; Kuilman et al., 2008, 2010; Kuilman and Peeper, 2009) and shown in Figure 4. The SMS can act not only locally, but also systemically. This secretome is largely composed of a number of extracellular growth factors, including TGF-β, EGF, PDGF, HGF, IGF1-binding proteins, as well as cytokines/chemokines, receptors antagonists, and receptor decoys and extracellular-matrix-remodeling proteins (Freund et al., 2010). These SMS components can fortify senescence and induce senescent phenotype in other cells in an autocrine and paracrine manner (Kuilman et al., 2010; Acosta et al., 2013) setting a stage for an efficient communication between senescent cells and their environment and depicted in Figure 4. Quintessential to cellular communication is an ability of a cell to secrete a protein that is able of diffusion through the extracellular environment to the neighboring cells in order to bind a cell surface receptor. This type of cell-to-cell communication is called *paracrine* (Lauffenburger et al., 1987). Pro-senescence effects could be also imposed in non-cell-autonomous fashion (paracrine) (Figure 4, center). Upon ligand-receptor binding, a wide range of biochemical and biophysical changes inside the receptor cell could trigger various cellular responses (Lauffenburger et al., 1987). In addition, a ligand can also bind to receptors on the surface of the same cell that produces the ligand in cell-autonomous fashion. This type of communication called *autocrine* (Figure 4, left side).

Although initially described in fibroblasts (Coppe et al., 2008; Rodier et al., 2009) as a tumor preventive mechanism, SMS is not restricted to the fibroblast. Importantly, different cells types, including MSC, upon senescence broadcast different SMS, and these broadcasts could vary in accordance to the different triggers that induce senescence and in response to the micro-niche that host senescent cells (Table 1). Remarkably, detailed analysis of the different SMS revealed that broadcast from senescent cells could exert opposing and contradictory effects shown in Figure 3. On one hand, SMS can prevent tumorigenesis by imposing senescence and promoting subsequent immunological clearance of cancer cells (Xue et al., 2007; Kang et al., 2011; Raouf et al., 2015). On the other hand, SMS, paradoxically, can potentiate the tumorigenic properties of cancer cells (Krtolica et al., 2001; Liu and Hornsby, 2007; Yoshimoto et al., 2013).

**Table 1:** SMS composition in different models of senescence.

**SMS** **Replicative, genotoxic, and OIS** **Lasry and Ben-Neriah, 2015**	**SIR** **Genotoxic** **Lasry and Ben-Neriah, 2015**	**SMS** **Genotoxic** **Gaur et al., 2017b**
***BMP6***		***BMP6***
***CCL1***		***CCL1***
***CCL8***		***CCL8***
***Eotaxin 3***		***Eotaxin 3***
***FAS***		***FAS***
***HGF***		***HGF***
***ICAM-1***	***ICAM-1***	***ICAM-1***
***IGF-1***		***IGF-1***
	***IGFBP4***	***IGFBP4***
***IL-6***		***IL-6***
***IL-13***		***IL-13***
***IL-15***		***IL-15***
***NAP2***		***NAP2***
***TGFb1***		***TGFb1***
Activin A	Atf5	CCL5
Amphiregulin	Ccdc33	CCL17
Angiogenin	CD9	CCL27
Axl	CD14	CSF1
bFGF	CD276	CXCL11
BMP2	CD40	Eotaxin 2
CCL2	Cpa2	FGF-6
CCL3	CXCL1	GDNF
CCL7	CXCL3	ICAM-2
CCL13	CXCL5	IL-3
CCL16	CXCL9	IL-5
CD9	CXCL10	IL-10
CD55	ETV5	IL-16
CSF2	Faim2	L-selectin
CSF2RB	Fam129a	LAP
CXCL1	Hamp	Leptin
CXCL5	Hif3a	Leptin R
EGFR	IFIT1	PDGF AB
ENA78	IFIT2	PDGF BB
Epiregulin	IFIT3	PRL
Ets2	IFITM3	SCF
FGF7	IGF2BP1	TGFb2
GCP2	IGFBP1	TNFSF14
GDF 15	IL-1a	VEGF R3
GEM	IL-1f9	
GMCSF	IL-1RN	
Gmfg	ISG15	
Heregulin	Itga2	
ICAM-3	Lass3	
IGFBP-1	Lpo	
IGFBP-2	Mapk11	
IGFBP-6	Mif	
IGFF-2R	Msx2	
IL-1a	MX1	
IL-1b	MX2	
IL-7	NXN	
Inhibin A	OAS2	
IQGAP2	OAS3	
Itga2	OLR1	
Itpka	Phlda1	
Jun	Pla2g2a	
Mif	Pla2g2f	
MMP1	Prss22	
MMP2	PTGES	
MMP3	Rel	
MMP10	Runx1	
Osteoprotegerin	SLC7a11	
PAI1	Sox17	
Pecam1	Sox4	
PIGF	Tirap	
PTGES	TLR1	
RPS6ka5	TLR2	
Timp2	TNFRSF19	
uPAR	TNFRSF8	
VEGFa	Tnip2	
VEGFc	USP18	
Wnt2	Xaf1	

*Comparison of previously reported SMS (Lasry and Ben-Neriah, 2015) to SMS of adipose-derived mesenchymal stem cells induced to acute senescence by genotoxic stress. Column one lists SMS of normal endothelial, epithelial and fibroblast cells reported in Coppe et al. (2008), Kuilman et al. (2008) and Acosta et al. (2013). Column two shows the senescence-associated inflammatory responses (SIR) from intestinal cells (Pribluda et al., 2013). The third from the left column lists SMS from adipose-derived mesenchymal stem cells induced to senescence by bleomycin (Gaur et al., 2017b). Common proteins are highlighted in bold red. Atf5, Activating Transcription Factor 5; Axl, AXL Receptor Tyrosine Kinase; bFGF, Basic Fibroblast Growth Factor; BMP2, Bone Morphogenetic Protein 2; BMP6, Bone Morphogenetic Protein 6; Ccdc33, Coiled-Coil Domain Containing 33; CCL1, Chemokine (C-C Motif) Ligand 1; CCL2, Chemokine (C-C Motif) Ligand 2; CCL3, Chemokine (C-C Motif) Ligand 3; CCL5, Chemokine (C-C Motif) Ligand 5; CCL7, Chemokine (C-C Motif) Ligand 7; CCL8, Chemokine (C-C Motif) Ligand 8; CCL13, Chemokine (C-C Motif) Ligand 13; CCL16, Chemokine (C-C Motif) Ligand 16; CCL17, Chemokine (C-C Motif) Ligand 17; CCL27 (CTACK), C-C Motif Chemokine Ligand 27; CD14, CD14 Molecule, Myeloid Cell-Specific Leucine-Rich Glycoprotein; CD276, CD276 Molecule; CD40, CD40 Molecule; CD55, CD55 Molecule; CD9, CD9 Molecule; Cpa2, Carboxypeptidase A2; CSF2, Colony Stimulating Factor 2; CSF2RB, Colony Stimulating Factor 2 Receptor Beta; CXCL1, C-X-C Motif Chemokine Ligand 1; CXCL3, C-X-C Motif Chemokine Ligand 3; CXCL5, C-X-C Motif Chemokine Ligand 5; CXCL9, C-X-C Motif Chemokine Ligand 9; CXCL10, C-X-C Motif Chemokine Ligand 10; CXCL11, C-X-C Motif Chemokine Ligand 11; EGFR, Epidermal growth factor receptor; ENA78, Epithelial-Derived Neutrophil-Activating Protein 78; Ets2, ETS Proto-Oncogene 2, Transcription Factor; ETV5, ETS Variant 5; Faim2, Fas Apoptotic Inhibitory Molecule 2; Fam129a, Family With Sequence Similarity 129 Member A; FAS, Fas Cell Surface Death Receptor; FGF-6, Fibroblast Growth Factor 6; FGF-7, Fibroblast Growth Factor 7; GCP2, granulocyte chemotactic protein 2; GDF 15, Growth Differentiation Factor 15; GDNF, Glial Cell Derived Neurotrophic Factor; GEM, GTP Binding Protein Overexpressed In Skeletal Muscle; GMCSF, Granulocyte-macrophage colony-stimulating factor; Gmfg, Glia Maturation Factor Gamma; Hamp, Hepcidin Antimicrobial Peptide; HGF, Hepatocyte growth factor; Hif3a, Hypoxia Inducible Factor 3 Alpha Subunit; I-309 (CCL1), T Lymphocyte-Secreted Protein I-309**;** I-TAC (CXCL11), Interferon-Inducible T-Cell Alpha Chemo-attractant; ICAM-1, Intercellular Adhesion Molecule 1; ICAM-2, Intercellular Adhesion Molecule 2; ICAM-3, Intercellular Adhesion Molecule 3; IFIT1, Interferon Induced Protein With Tetratricopeptide Repeats 1; IFIT2, Interferon Induced Protein With Tetratricopeptide Repeats 2; IFIT3, Interferon Induced Protein With Tetratricopeptide Repeats 3; IFITM3, Interferon Induced Transmembrane Protein 3; IGF-1, Insulin-like growth factor; IGF2BP1, Insulin Like Growth Factor 2 MRNA Binding Protein 1; IGFBP-1, Insulin Like Growth Factor Binding Protein 1; IGFBP-2, Insulin Like Growth Factor Binding Protein 2; IGFBP-4, Insulin Like Growth Factor Binding Protein 4; IGFBP-6, Insulin Like Growth Factor Binding Protein 6; IGFF-2R, Insulin Like Growth Factor 2 Receptor; IL-1a, Interleukin 1 alpha; IL-1b, Interleukin 1 beta; IL-3, Interleukin 3; IL-5, Interleukin 5; IL-6, Interleukin 6; IL7, Interleukin 7; IL-10, Interleukin 10; IL-13, Interleukin 13; IL-15, Interleukin 15; IL-1f9, Interleukin-1 Family Member 9; IL-1RN, Interleukin 1 Receptor Antagonist; IL-16, Interleukin 16; IQGAP2, IQ Motif Containing GTPase Activating Protein 2; ISG15, Interferon-Stimulated Protein, 15 KDa, Ubiquitin-Like Modifier; Itga2, Integrin Subunit Alpha 2; Itpka, Inositol-Trisphosphate 3-Kinase A; Jun, Jun Proto-Oncogene, AP-1 Transcription Factor Subunit; LAP (TGFb1), latency-associated peptide; Lass3, LAG1 Longevity Assurance Homolog 3; Leptin R, Leptin receptor; LIGHT (TNFSF14), Tumor Necrosis Factor Superfamily Member 14; Lpo, Lactoperoxidase; M-CSF (CSF1), Macrophage Colony Stimulating Factor 1; Mapk11, Mitogen-Activated Protein Kinase 11; MCP-2 (CCL8), Monocyte Chemoattractant Protein 2; Mif, Macrophage Migration Inhibitory Factor; MMP1, Matrix Metallopeptidase 1; MMP2, Matrix Metallopeptidase 2; MMP3, Matrix Metallopeptidase 3; MMP10, Matrix Metallopeptidase 10; Msx2, Msh Homeobox 2; MX1, MX Dynamin Like GTPase 1; MX2, MX Dynamin Like GTPase 2; NAP2 (PPBP), Neutrophil-Activating Peptide 2, Platelet Basic Protein; NXN, Nucleoredoxin; OAS2, 2′-5′-Oligoadenylate Synthetase 2; OAS3, 2′-5′-Oligoadenylate Synthetase 3; OLR1, Oxidized Low Density Lipoprotein Receptor 1; PAI1, Phenylalanine ammonia-lyase 1; PDGF AB, Platelet Derived Growth Factor Subunit A beta; PDGF BB, Platelet Derived Growth Factor Subunit b beta; Pecam1, Platelet And Endothelial Cell Adhesion Molecule 1; Phlda1, Pleckstrin Homology Like Domain Family A Member 1; PIGF, Phosphatidylinositol Glycan Anchor Biosynthesis Class F; Pla2g2a, Phospholipase A2 Group IIA; Pla2g2f, Phospholipase A2 Group IIF; PRL, Prolactin; Prss22, Protease- Serine 22; PTGES, Prostaglandin E Synthase; RANTES (CCL5), Regulated Upon Activation, Normally T-Expressed, And Presumably Secreted; Rel, REL Proto-Oncogene, NF-KB Subunit; RPS6ka5, Ribosomal Protein S6 Kinase A5; Runx1, Runt Related Transcription Factor 1; SCF, Stem cell factor; SLC7a11, Solute Carrier Family 7 Member 11; Sox17, SRY-Related HMG-Box Transcription Factor SOX17; Sox4, SRY (Sex Determining Region Y)-Box 4; TARC (CCL17), Thymus And Activation-Regulated Chemokine; TGFb1, Transforming Growth Factor Beta 1; TGFb2, Transforming Growth Factor Beta 2; Timp2, TIMP Metallopeptidase Inhibitor 2; Tirap, TIR Domain Containing Adaptor Protein; TLR1, Toll Like Receptor 1; TLR2, Toll Like Receptor 2; TNFRSF19, Tumor necrosis factor receptor superfamily 19; TNFRSF8, Tumor necrosis factor receptor superfamily 8; Tnip2, TNF Alpha Induced Protein 3 interactive protein 2; uPAR, Plasminogen Activator, Urokinase Receptor; USP18, Ubiquitin Specific Peptidase 18; VEGF R3, Vascular Endothelial Growth Factor receptor 3; VEGFa, Vascular Endothelial Growth Factor A; VEGFc, Vascular Endothelial Growth Factor C; Wnt2, Wingless-Type MMTV Integration Site Family Member 2; Xaf1, X-Linked Inhibitor Of Apoptosis Associated Factor 1*.

To add more complexity, it has been demonstrated that SMS can also promote regeneration through the induction of the cell plasticity and stemness (Ritschka et al., 2017). Taken together this suggests more complex physiological roles of SMS than are currently understood. To patch things up between seemingly opposing influences of the cellular senescence outcomes (beneficial and detrimental shown in Figure 3) has been an enormous challenge. Next, we will take a closer look at the different aspects of SMS.

### Autocrine SMS to reinforce senescent state

In autocrine fashion senescent cells activate a self-amplifying SMS network in which CXCR2-binding chemokine reinforces growth arrest. Specific components of the SMS such as IGFBP-7 (Wajapeyee et al., 2008), PAI-1 (Kortlever et al., 2006), IL-6 and CXCR2-binding chemokines (such as IL-8 or GROα) (Acosta et al., 2008) can reinforce senescence (Figure 3, right side shown in pink and Figure 4, left). Since the components of the DNA-damage response are essential to both replicative and OIS, up-regulation of CXCR2 increases DNA damage, albeit it is currently unclear how CXCR2 activity influences the DDR and p53. It has been hypothesized that the mechanism might be linked to the increase in ROS levels that ultimately can drive OIS (Lee et al., 1999) and DNA damage at the ends of the telomers (Passos et al., 2007). It has been suggested that, CXCR2 regulation of senescence is driven by secretion of IGFBP-7, which impacts on MAPK signaling (Wajapeyee et al., 2008) or *via* regulation of PI3-kinase pathway by other secreted factors such as PAI-1 (Kortlever et al., 2006).

Taken together, these data suggest the existence of a positive feedback loop involving chemokine and receptor signaling that acts to reinforce senescence.

### Paracrine SMS to transmit senescent state to the normal cells

It has also been demonstrated that senescence as a state, could extend the “footprint” of growth arrest to normal or pre-malignant neighboring cells (Figure 4, right side). Experiments with OIS cells indicate that senescence state can be spread around in non-cell autonomous manner. Remarkably, the paracrine effect (local or systemic) of senescent cells can trigger and reinforce senescence in the neighboring cells encompassing tissue or organ. Paracrine senescence resembles a full senescence response, which is characterized by oxidative damage to DNA and cellular proteins, an efficient cell cycle arrest *via* activation of Rb/p16^Ink4a^ and p53/p21^Cip1^ pathways and marked by an amplified production of IL-8 cytokine. The paracrine senescence can be transmitted by ligands of TGF-β and BMP branches that mediate changes in the transcriptional program through the SMAD family members SMAD2/3 and SMAD1/5 (Acosta et al., 2013). These ligands include TGF-β1, BMP6, BMP2, Inhibin A, VEGF, CCL2, CCL20, and GDF15 (Acosta et al., 2013).

However, not all cells surrounding senescent cells undergo paracrine senescence (Figure 4, right side). Levels of soluble factors, gradients of their concentration, as well as an ability of different cell types to read such transmitted SMS, would influence whether or not cells undergo paracrine senescence *in vivo*. In addition and important to the subject of this discussion is a notion that a strength of both autocrine and paracrine cell communication are reciprocal to the time and quantitates of secreted signals, as well as a length of the exposure to the signal (Berezhkovskii et al., 2008). The SMS, as any communication system, is not static but a rather well controlled and dynamic process. It is highly probable that SMS signals vary with time and include not only an autocrine feedback loop but also an ability of senescent cells to “listen” to the environment and respond to it by adjusting or spatially restricting SMS secretory output (schematically shown in Figure 3 and discussed in details in Kuilman and Peeper, 2009).

### SMS: regeneration, inflammasome, and inflammation

Timely and controlled exposure of the microenvironment to SMS may be beneficial and directly promote regeneration (Ritschka et al., 2017) and shown in Figure 3. The new line of evidence demonstrates that primary mouse keratinocytes transiently exposed to the SMS exhibit upregulated expression of stem cell markers and boost regenerative capacity *in vivo*. Induction of senescence in a single liver cell has shown to induce an increase in the stem cell markers in the surrounding tissue of the liver. This experimental evidence suggests an important biological function of senescence in the molecular underpinning of the organ and tissues regenerative program. Such senescence promoting cell plasticity is very similar to previously discussed developmental senescence (Munoz-Espin et al., 2013; Storer et al., 2013; Mosteiro et al., 2016). Teasing apart functional influences of the well-known potent mediator of inflammation, NF-κB, on SMS broadcast, a number of new studies revealed the mechanistic aspects of its beneficial influences during senescence. The controlled NF-κB-mediated inflammation processes might be indispensable in regulating normal tissue development and regeneration. It has been shown that senescence and local controlled inflammation contributes to hair follicle stem cell regeneration upon skin wounding (Chen C. C. et al., 2015; Gaur et al., 2017a), and drives the development of the hair follicle (Schmidt-Ullrich et al., 2001; Zhang et al., 2009). Nonetheless, much longer exposure to the SMS results in a cell-intrinsic senescence arrest to counteract with carrying forward the regenerative stimuli (Ritschka et al., 2017).

NF-κB is also known as a potent mediator of inflammation upon aging and tumorigenesis. It is tempting to speculate that prolonged exposure to SMS also could result in an over-exuberant expansion of stem cells, which, when misregulated, can increase the likelihood of mutagenesis in these cells. Also, such over-exuberant stem cell expansion might favor the situation when plasticity of expanded stem cells and their under-differentiated state create an environment highly receptive to carcinogenic transformation (Tschaharganeh et al., 2014; Varga et al., 2014). Therefore, it is very likely that senescent cells act as niche-like signaling centers, where SMS produced by senescent cells induces plasticity and stemness in neighboring cells while imposing blocks on their own proliferation.

**Figure 5:**
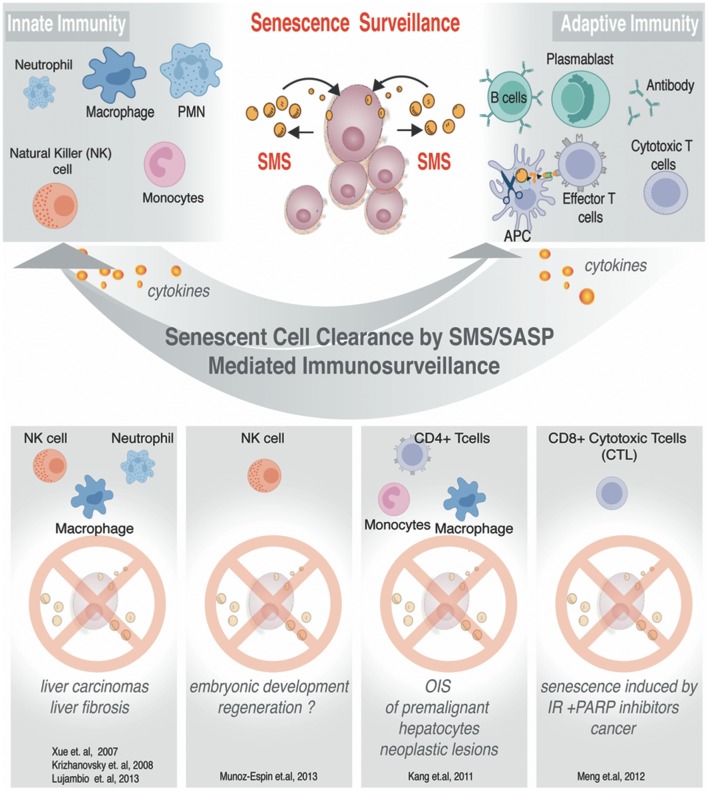
SMS-mediated immunosurveillance. Senescent cells can impose regulations on innate and adaptive immune cells. Known examples of senescent cells clearance. Immune effector cells are listed to illustrate condition-specific senescent cells clearance.

In an unperturbed setting, SMS activates the immune system to promote clearance of the senescent cells broadcasting SMS (shown in Figure 5). Such designated “senescence surveillance” was initially successfully demonstrated in the mouse livers models of OIS obtained by stable, transposon-mediated transfer of oncogenic *N-ras* (Nras^G12V^), where senescent hepatocytes undergo immune-mediated clearance (Xue et al., 2007; Acosta et al., 2008; Kang et al., 2011). Such clearance of the senescent cells will leave the organ or tissue with a cell deficit; therefore it aligns with a logical concept why SMS functions to ensure cellular plasticity to restore homeostasis and regeneration while inducing the immune system to liquidate all of the damaged senescent cells within the senescent-niche-like center.

It is important to discuss specific signal transduction pathways affected by SMS. Several of these pathways are reviewed in detail (Kuilman and Peeper, 2009). In the context of this review, we will focus on the emerging importance of an inflammasome in senescence, leaving out of the picture an inflammasome activation by infections agents (Rathinam and Fitzgerald, 2016; Sharma and Kanneganti, 2016). Currently, the mechanistic pathways that link an inflammasome to cellular stresses and cellular senescence are unknown. Excitingly, it was demonstrated that the inflammasome and IL-1 signaling impose inflammasome-mediated controls over SMS and senescent cells inflammatory output. The focus of IL-1α signaling revealed that IL-1α is a potent inducer of multiple SMS components (Gardner et al., 2015) and suggested that IL-1 has probably a more prominent role than TGF-β signaling in controlling the SMS (Acosta et al., 2013).

Senescent cells secrete out mature forms of both IL-1α and IL-1β, implying possible activation of the inflammasome components. The processing of inactive precursors for both of these factors does irrevocably depend on the functional multiprotein inflammasome complex, which incorporates together with multiple adapter molecule and enzymatic activity of Caspase 1 (Schroder and Tschopp, 2010; Strowig et al., 2012). Published data demonstrate that ectopic IL-1α expression triggers cell cycle arrest in IMR90 fibroblasts cells causing senescence (Acosta et al., 2013). The senescence phenotype is characterized by the high level of DNA and oxidative damage, as well as by proliferative arrest via p53 and p21^Cip1^.At variance with this observation, knockdowns of the IL-1 receptor, as well as some of the components of inflammasome machinery could to a certain degree prevent OIS (Acosta et al., 2013). These results highlight the direct involvement of inflammasome in the regulation of senescence and add more complexity to the pro- and antitumor effects of the cellular senescence.

Although numerous attempts have been made in order to frame the blueprint of inflammasome and its architecture, the structural features of the inflammasome in intact cells remains unresolved. Less is known about details of the inflammasome-mediated process in the senescent cells. Beyond driving conventional pyroptosis (inflammatory cell death) and maturation of the cytokines, the inflammasome activation also advances the release of ASC (apoptosis-associated speck-like protein containing a caspase activation and recruitment domain) specks that are efficiently phagocytosed by other cells in the neighborhood. The ASC nucleation, and the speck release in the extracellular medium act as the inflammasome-perpetuating signal when consumed by recipient cells (Baroja-Mazo et al., 2014; Franklin et al., 2014). Numerous nucleation events might allow the ASC speck to act as a scaffold for the addition of soluble ASC monomers, *via* a *prionic*, self-propagating mechanism (Cheng et al., 2010; Cai et al., 2014; Franklin et al., 2014). It remains to be investigated whether ASC participates in senescence propagation shown in Figure 4. Interestingly, the ASC speck could not only boost an inflammasome response to amplify the inflammation, but also by acting upstream of multiple molecular and cellular signals, represents a powerful sensor that could maintain the balance between inflammatory response and its resolution (Rathinam and Fitzgerald, 2016; Sharma and Kanneganti, 2016).

Recent studies, however, are just beginning to unveil the structural and functional composition of the inflammasome machinery. It is appealing to speculate that the inflammasome function extends beyond its influence in cell-autonomous and paracrine mechanisms of cellular senescence to the ability of inflammasome to regulate both innate and adaptive immune response *via* SMS.

### Communication with immunosystem

As we discussed above, the SMS provides senescent cells with diverse functionality, among which is an important property of senescence cells—the ability to alert the immune system. The SMS can also evoke a local inflammatory response with complex effects, among which is the recruitment of inflammatory cells. The recruitment of the inflammatory cells has shown to result in senescent cells removal leading to tissue remodeling as well as regeneration (Krizhanovsky et al., 2008; Freund et al., 2010; Kang et al., 2011; Hoenicke and Zender, 2012). While detailed reviews on how senescent cells can communicate with the immune system can be found elsewhere (Hoenicke and Zender, 2012), we have summarized some of the best-studied examples in Figure 5. In this chapter, we will focus in greater detail on the SMS related to MSC, and discuss how this system can influence functional outcome both in a natural setting and upon applications of MSC-based therapeutic strategies in a clinic.

The primary trophic property of MSC is secretion of mitogenic growth factors such as TGF-α and TGF–β, basic vascular endothelial growth factor (VEGF), hepatocyte growth factor (HGF), epithelial growth factor (EGF), fibroblast growth factor (FGF-2) and insulin-like growth factor-1 (IGF1). Systemically, all of these factors have shown to increase fibroblasts, epithelial and endothelial cell division or differentiation (Holgate et al., 2000; O'cearbhaill et al., 2008; Bai et al., 2012; Murphy et al., 2013; Chen et al., 2014). These observations provide evidence that the MSC-triggered cellular communication circuitry is necessary for tissue or organ remodeling and regeneration. In addition, MSC in their natural environment secrete both pro-and anti-inflammatory cytokines as well as their antagonists (Spees et al., 2016). Such secretions have shown to provide sophisticated signaling guidance to many cells of the immune system, such as dendritic cells, macrophages, natural killer cell, monocytes, as well as B and T lymphocytes (Uccelli et al., 2008; Ichim et al., 2010; Yi and Song, 2012; Murphy et al., 2013; Ben-Ami et al., 2014). The phenomenon is poorly understood on the molecular level, but conceptually explains many successful applications of MSC in the clinic related to injury repair and improvement of tissue homeostasis.

On the other hand, numerous reports have demonstrated that exposure of MSC to pro-inflammatory cytokines in their microenvironment and *ex vivo* increases the migratory capacity of human MSC (Niu et al., 2015), as well as induces the production of chemokines and chemotactic factors that permit MSC to either suppress immune reactions (Aggarwal and Pittenger, 2005; Glennie et al., 2005; Ren et al., 2008), or restore immunity as was described recently in the case of mucosal immunosenescence in elderly (Tsuruhara et al., 2017). The best-documented immune-modulatory effect of MSC is their ability to impose G0/G1 phase arrest in the activated T cells, thus inhibiting T cell proliferation (Aggarwal and Pittenger, 2005; Glennie et al., 2005; Benvenuto et al., 2007; Xue et al., 2010). The exact mechanistic aspects by which this modulation occurs are only partially understood and seemingly contradictive. This is, in part, due to lack of data that makes an assessment of how MSC senescence both *in vivo* or *ex vivo* could contribute to the immunomodulatory properties.

In parallel with other cellular models, different modes of MSC senescence might considerably alter the composition of MSC secretome (Capasso et al., 2015; Ozcan et al., 2016; Gaur et al., 2017b) resulting in impairment or enhancement of the key MSC biological functions.

Interestingly, the secretion of a wide array of inflammatory molecules, such as interleukin-1 (IL-1), IL-2, IL-12, tumor necrosis factor-alpha (TNF-α) and interferon-gamma (INF-γ) (see for review Murphy et al., 2013) can affect complex signaling guidance to many inflammatory cells including those that ultimately participate in the senescence cells clearance (as shown in Figure 5). Also, one can envision that through the negative feedback loop, SMS of MSC might influence and/or modulate the senescent cells-generated micro-niche leading to exacerbated or suppressed immune responses within tissues or organs.

## SMS/SASP components and their role in immunomodulation

The SMS of senescent cells is complex. The molecular underpinnings in the heart of regulation of immune surveillance by senescent cells are not completely understood. However, it has been demonstrated that hepatocytes upon OIS can attract cells of innate and adaptive immune system through the specialized secretion of senescence-associated chemokines and cytokines (Kang et al., 2011; Figure 5). Detailed analysis of this model allowed to exclude direct CD4+ T cell cytotoxicity, by phenotypic identification of Th-1-type prevalence in the population of CD4+ T cell. Th-1-type response stimulate monocytes and freshly replenished macrophages to clearance of senescent hepatocytes (Figure 5). The resident macrophages (Kupffer cells) in the liver have been shown to take no blame in the elimination of Nras ^G12V^ -expressing senescent hepatocytes (Kang et al., 2011). Collectively, these data showed that CD4+ T cell-mediated immune clearance of premalignant senescent cells is crucial to suppress liver cancer development and that OIS plays an important role in the induction of these specialized immune responses. Similarly, it has been demonstrated that SMS from senescent cells induced by ionizing radiation (IR) combined with the poly (ADP-ribose) polymerase inhibitor (PARPi) *veliparib* deployed to inhibit DNA repair can activate cytotoxic CD8+ T lymphocytes (CTLs), which in turn mediate an effective antitumor response by clearing senescence cells and shrinking tumor size (Meng et al., 2012). Natural killer cells (NK) were also demonstrated to be able to clear senescent cells under specific SMS in order to ameliorate and or restrict fibrosis progression (Krizhanovsky et al., 2008; Sagiv et al., 2016). Along the line of these investigations, Pitiyage et al. (2011) have demonstrated that by a telomere-independent mechanism, senescent MSC had been accumulated in human oral submucous fibrosis and these senescent cells participated in amelioration of the fibrotic tissue through the secretion of matrix metalloproteinases (Pitiyage et al., 2011).

In the context of these emerging studies of the significance of immune surveillance triggered by senescent cells in development and disease pathology, it is critical to discuss how these very distinct immune responses could be orchestrated by senescent cells (Figure 5). Next, we will take a closer look at the components of SMS produced by MSC (Table 1) that could be involved in mediating different immunomodulatory responses.

### Fas and fas ligands

Fas (CD95/Apo-1) is a transmembrane protein belonging to the TNF/nerve growth factor receptor superfamily. Fas binds to its natural ligand FasL (CD178) (Voss et al., 2008). The Fas/FasL system regulates several processes of the immune system, such as T cell selection in the thymus for the acquisition of self-tolerance (Xing and Hogquist, 2012), clonal deletion of activated T cells to maintain T cell homeostasis and to downregulate inflammatory processes (Gronski and Weinem, 2006). The studies performed in mutant mouse models, Fas-mutant *(lpr)* and FasL-deficient *(gld)*, have shown lymphoproliferative changes, thus suggesting that Fas/FasL system is required for suppression/regulation of activated effector T cells (Salmaso et al., 2002; Beeston et al., 2010). Fas/FasL system is also involved in the maintenance of T cells immune regulation and homeostasis by influencing CD4+FoxP3+ T regulatory cells (T_reg_). Fas/FasL could moderate a development of tolerogenic DCs and T_reg_, therefore accelerating a suppression of the effective immune response (Bien et al., 2016). In addition, Fas and its ligand FasL are able to regulate activated B cells (Jacobson et al., 1996) in order to establish “immune-privileged” sites (Abrahams et al., 2003), thus resulting in better host vs. graft rejection outcomes.

### L- and E-selectins

Selectins belong to a family of type I transmembrane carbohydrate-binding glycoproteins and are expressed on leukocytes, platelets, and endothelial cells, and are essential for these immune cells trafficking (Ley and Kansas, 2004; Nolz and Harty, 2011). The selectins and their ligands are implicated in several physiological processes such as leukocyte adhesion, transmigration, homing during chronic and acute inflammation and also during oncogenesis (Ley, 2003). It is accepted that selectin's family functions in the biological pathways responsible for maintaining the cell- mediated adaptive antitumor immunity and immune surveillance following activation of naive lymphocytes in tumor draining lymph nodes and tumor sites.

L-Selectin (CD62L), one of the three members of the selectin family, is expressed on granulocytes, monocytes and lymphocytes and performs a vital function in maintaining such immune surveillance, by directing naive lymphocytes to peripheral lymph nodes for subsequent activation upon antigen interaction (Ley and Kansas, 2004; Watanabe et al., 2008). Normal T cells responses, as well as the homing of naive T cells to lymph nodes, is highly dependent on the normal levels of L-Selectin (Bradley et al., 1994; Stremmel et al., 2006; Hanson et al., 2009).

E-Selectin or (CD62E) is expressed in non-inflamed skin endothelium and become present on endothelium cells of most organs upon activation (Keelan et al., 1994; Kansas, 1996). This protein is involved in leukocytes tethering, rolling, cell signaling and chemotaxis (Ley and Kansas, 2004; Barthel et al., 2007; Nolz and Harty, 2011). Similar to L-Selectins, E-Selectin also has an amino-terminal domain for lectin binding, an epidermal growth factor (EGF)-like domain and short consensus repeats (SCRs) that have a role in the adhesion process (Jutila et al., 1992). For a successful immune response, all 3 selectins (L-, P-, and E-Selectin), along with other adhesion molecules are required to activate the cascade of events leading to leukocyte binding and migration (Hogg and Landis, 1993).

### ICAM-1

Adhesion molecules, such as intracellular adhesion molecule-1 (ICAM-1), is among those overexpressed in the MSC's SMS. ICAM-1 is a member of the immunoglobulin superfamily (Springer, 1994) and its membrane-bound isoform is widely known for its functions in adhesion and trafficking of immune cells across the blood vessels during all inflammatory responses (Marlin and Springer, 1987; Springer, 1994; Ley and Tedder, 1995). ICAM-1 is expressed on a wide range of immune cells such as monocyte-macrophage lineage cells, B-lymphocytes, plasma cells and on activated and memory T cells where it functions to mediate immune synapses thereby initiating the signaling pathways for major histocompatibility complex (MHC-I and MHC-II) molecules. However, it also exists in the soluble form, sICAM-1. Soluble ICAM-1 interacts with lymphocyte function associated antigen-1 (LFA-1) molecules, and therefore, the production of sICAM-1 is thought to have immunomodulatory consequences. The experiments in sICAM transgenic animal models indicate that soluble ICAM impedes on cell-cell interactions mediated by ICAM and produces potent suppression of immune responses (Wang et al., 2005), thus suggesting the important role for this protein in SMS-mediated regulation of immunity.

### Leptin

Leptin, another factor secreted by SMS of MSC (Table 1), is an adipocyte-derived hormone, which has proven to have an important function in regulating energy metabolism, immunomodulation and hematopoiesis (Loffreda et al., 1998; Fantuzzi and Faggioni, 2000; Perez-Perez et al., 2017). Leptin is a member of the long-chain helical cytokine family that is comprised of IL-2, IL-6, IL-11, IL-12, and G-CSF (Peelman et al., 2004; Lam and Lu, 2007), and its expression has shown to be regulated by food intake, hormones, as well as inflammatory factors (Zhang et al., 1994; Iikuni et al., 2008; Andrade-Oliveira et al., 2015). The receptor for leptin (LEPR) is widely expressed on the immune cells, for example, monocytes, granulocytes and natural killer (NK) cells, and possesses signaling capabilities similar to IL-6 cytokine receptor in the activation of JAK-STAT, PI3K, and MAPK signaling cascades (Zabeau et al., 2003). Leptin functions as a modulator of both adaptive and innate immunity (Tian et al., 2002; Bernotiene et al., 2006). This protein affects innate immunity by regulating the function and immune activity of mast cells by increasing their survival rate and migration. It has also been implicated in the mediation of phagocytosis by monocytes/macrophages, and augmentation of migration of eosinophils as well as their survival. It promotes the inflammatory factors secretion such as chemokines and IL-1β, IL-6, IL-8. In adaptive immunity, the role for leptin is not yet fully explored. However, recent data point to the leptin's function in promoting generation and survival of T cells by reducing their apoptosis. Leptin promotes increased expression and secretion of interferon-γ (IFN-γ), thus stimulating the Th-17 development and responses. However, leptin could also act as a negative effector of proliferation/expansion of human regulatory T cells (T_reg_) (De Rosa et al., 2007) by a process that involves activation CDKI p27^Kip1^ pathway. These data, together with the ability of leptin to activate secretion of TNF-α, IL-6, and IL-10 in B- cells, suggests that this protein functions as a potent pro-inflammatory mediator relevant to numerous immunological outcomes in development and disease.

### IL-1RA

Interleukin-1 receptor antagonist (IL-1RA) is a ligand for interleukin-1 (IL-1) receptor family with significant anti-inflammatory property (Palomo et al., 2015). IL-1 receptor is an important mediator of immune and inflammatory responses and its activity is tightly regulated at multiple checkpoints, such as protein expression level, protein processing and its maturation. IL-1RA (antagonist) is produced by a number of cells of the immune system such as monocytes, macrophages, neutrophils, DCs, as well as non-immune cells such as epithelial cells and MSC. These cells also produce IL-1α or IL-1β for IL-1), with similar binding affinities. IL-1RA competes with IL-1α or IL-1β for binding to IL-1 receptor (IL-1R1) resulting in suppression of IL-1 activity and has been already approved for use in humans for the treatment of rheumatoid arthritis. Recently, it has shown that IL-1R1 acts as the regulator of Th-17/T_reg_ axis in mouse models. Mechanistically, IL-1RA is implicated in the Th-17/T_reg_ balance by decreasing the number of Th-17 cells while promoting the expansion of T_reg_ cells in the spleen (Spees et al., 2016). Its systemic administration has numerous beneficial properties including a recently reported ability to promote neurogenesis in the experimental models of stroke (Pradillo et al., 2017), thus suggesting the important role of this protein in the context of SMS.

### Sgp130

Gp130 is the type I transmembrane signal transducer protein gp130 (CD130) found in abundance in the MSC SMS (Table 1). IL-6 first binds to the membrane of the cells with exposed non-signaling α-receptor IL-6R (mbIL-6R). This complex of IL-6 and IL-6R then binds to two molecules of gp130 followed by downstream activation of JAK/STAT, ERK, and PI3K signals to arm IL-6-signal transduction pathways. In the immune system, the maintenance of CD4^+^ Th-17 cells occurs by direct involvement of IL-6 *trans-signaling*. An alternative splicing rather than limited proteolysis produces a soluble form of the signal transducer protein gp130 (sgp130) (Mullberg et al., 1993). Numerous data demonstrate that monomeric natural occurring soluble form of sgp130 acts as a potent inhibitor of IL-6 *trans-signaling* (Jostock et al., 2001). Chimeric designer sgp130Fc was shown to block migration of CD4+ Th-17 cells in induced peritonitis model (Jones et al., 2010), leukocytes accumulation in air pouch model of acute inflammation (Rabe et al., 2008) and local inflammatory responses in collagen-induced arthritis (Nowell et al., 2009). These discoveries indicate that sgp130 acts a natural inhibitor for IL-6 *trans-signaling* (Jostock et al., 2001) and also has a potent affect on the classical IL-6 signaling pathway (Garbers et al., 2011), therefore making this protein an attractive signaling component of SMS of MSC.

## Conclusions

The significance of cellular senescence during normal development as well as in age-related pathologies and cancer, has attained a remarkable level of scrutiny in recent years. Remarkably, in the last ten years we gained deeper knowledge related to the multifaceted influences of the p53 and Rb pathways in mechanistic underpinnings of senescent phenotypes establishment and maintenance. Despite this steady progress in probing the roles of the p53-Rb axis, there is still plenty of uncharted territory remainingwhen it comes to other mechanisms that might contribute to the senescent phenotype of MSC and biological and pathophysiological consequences of the autocrine and paracrine influences of senescent MSC on the tissues and organs homeostasis.

MSC mediate numerous therapeutic effects by promoting repair directly via differentiation into critical cell types or indirectly through the secretion of substances and the activation of endogenous mechanisms. The success of the MSC transplantation therapy may depend on a variety of factors, which importantly, might depend on the ability of these cells to undergo replicative, stress-induced and oncogene-induced cellular senescence in local micro-environment. SMS secreted by MSC might not only affect the ability of the transplanted material to migrate to specific organs, or interact with vascular endothelium and transmigrate across it, but also ultimately to communicate with the immune system to control for tissue and organ homeostasis. Additionally, many therapeutic approaches, particularly those that employ genotoxic stressors, such as irradiation and DNA damaging agents, might also influence the stromal environment within human pathologies. The role of MSC and their stress-induced senescence or TIS need to be better elucidated in the context of the broad spectrum of human malignancies. Since besides cell death, senescence can be induced in tumor cells and surrounding tissue during chemotherapy, radiotherapy and immunotherapy in autocrine and paracrine fashion, the bystander senescence effects of stromal or adult stem cells should be taken into account when developing drugs and therapeutic approaches to counteract with malignancies. In the light of their physiological functions, senescent MSC cells through their SMS may induce changes in the tissue microenvironment and, unless immunologically cleared, could both positively and negatively counteract with therapeutic approaches. A better understanding of senescence process itself will allow us to control and modulate the course of numerous MSC therapeutic treatments, thus ushering only beneficial effects of senescence rather than boosting its detrimental side, which in many ways, might evoke an adverse response to a broad spectrum of therapeutic end-points.

## Author contributions

VL and MG: Conceived and wrote the review; VL and MG: Generated figures; AA-O: Helped in preparation of the manuscript.

### Conflict of interest statement

The authors declare that the research was conducted in the absence of any commercial or financial relationships that could be construed as a potential conflict of interest.
